# Noise Reduction in Spur Gear Systems

**DOI:** 10.3390/e22111306

**Published:** 2020-11-16

**Authors:** Aurelio Liguori, Enrico Armentani, Alcide Bertocco, Andrea Formato, Arcangelo Pellegrino, Francesco Villecco

**Affiliations:** 1 Degree Course in Transport, University “G. Fortunato”, Viale Raffaele Delcogliano, 12, 82100 Benevento, Italy; A.Liguori@Unifortunato.Eu; 2 Department of Chemical, Materials and Production Engineering, University of Naples “Federico II”, Piazzale V. Tecchio, 80, 80125 Napoli (NA), Italy; enrico.armentani@unina.it (E.A.); alcide.bertocco@unina.it (A.B.); 3 Department of Agricultural Science, University of Naples “Federico II”, Via Università 100, 80045 Portici (NA), Italy; 4 Department of Industrial Engineering, University of Salerno, Via Giovanni Paolo II, 132, 84084 Fisciano, Italy; apellegrino@unisa.it (A.P.); fvillecco@unisa.it (F.V.)

**Keywords:** noise reduction, gearboxes, entropy, coupled Eulerian–Lagrangian analysis

## Abstract

This article lists some tips for reducing gear case noise. With this aim, a static analysis was carried out in order to describe how stresses resulting from meshing gears affect the acoustic emissions. Different parameters were taken into account, such as the friction, material, and lubrication, in order to validate ideas from the literature and to make several comparisons. Furthermore, a coupled Eulerian–Lagrangian (CEL) analysis was performed, which was an innovative way of evaluating the sound pressure level of the aforementioned gears. Different parameters were considered again, such as the friction, lubrication, material, and rotational speed, in order to make different research comparisons. The analytical results agreed with those in the literature, both for the static analysis and CEL analysis—for example, it was shown that changing the material from steel to ductile iron improved the gear noise, while increasing the rotational speed or the friction increased the acoustic emissions. Regarding the CEL analysis, air was considered a perfect gas, but its viscosity or another state equation could have also been taken into account. Therefore, the above allowed us to state that research into these scientific fields will bring about reliable results.

## 1. Introduction

Under working conditions, spur gears are simultaneously subjected to mechanical and thermal loads, both of which are strictly related to noise emissions. Specifically, the transmitted torque affects the maximum stress on the teeth, rotational speed affects the cyclic loading, and friction leads to increased temperature in the gears and surrounding air. In [1], the interactions between gears and the air is considered, whereby the modeling air is treated as a one-dimensional ideal gas with constant entropy (ds = 0) and an adiabatic flow. The unification of the laws of thermodynamics and Newtonian mechanics has been pursued by many scientists in the last century. The principles are based on using entropy as a bridge between mechanics and thermodynamics [2]. Mechanothermodynamics (MTD) combines two branches of physics—mechanics and thermodynamics—to study the evolution of complex systems. For example, the theory of elasticity assumes that there is no entropy generation at the material level [3]. As a result, everything is reversible, which violates the second law of thermodynamics. The above approaches and models for the energy and stress–strain states of complex systems under thermodynamic and mechanical loads are considered in [4,5,6]. Damage and entropy concepts are important for building a model of an MTD system. Entropy-based models are often used for damage detection in cycling and creep load conditions [7,8,9]. Additionally, acoustic signals are often used for damage and fault detection [10,11]; therefore, noise and entropy should be related.

When gears work, especially under high loads and speeds, the noise and vibration caused by the rotation of the gears can be unbearable for people [12,13,14]. Even if it is possible to use some countermeasures against noise on the gears after they have been designed and built, in the design phase of a gearbox, predicting all of the noise reduction factors related to meshing and components—such as the supports, the transmission shafts, and the cases—is always preferred. In fact, transmission shafts, bearings, and their respective cases affect the acoustic emissions in a significant way, and they can be monitored during the design phase [15,16,17]. Meshing errors resulting from tolerances create an oscillation motion overlaying the uniform rotation of the mechanisms. These oscillations generate dynamic loads on the teeth acting on the transmission shaft, which deflect and consequently create dynamic loads on the bearings, meaning that gear case is excited and produces noise. If the natural vibration frequencies of the shaft and the case are equal to those of the gears, these two components enter into resonance and there is a remarkable amplification of noise [18,19,20,21,22]. Therefore, during the design phase, it is necessary to act on every component involved in the generation of noise. It is possible to achieve reductions in noise in different ways, such as by improving the kinematic precision of the system and by increasing the contact ratio so that dynamic loads on the teeth are reduced.

After minimizing the excitation resulting from the transmission device, it is possible to consider the dynamic loads acting on bearings. These can be reduced by changing the response of the support shaft system. In fact, transmission shafts can be stiffened so that their deflection is reduced and the teeth alignment improves. These changes remarkably reduce the noise of the gear device and improve the durability of bearings and other linked components. The dynamic response of the case can be changed by stiffening its internal or external surface and increasing its thickness [23,24,25]. It has to be taken into account that the teeth profiles can be changed to avoid interferences resulting from the deflection of shafts, bearings, and cases during meshing and separation phases. If these interferences are not balanced by some changes in the teeth profile, the load capacity of the gears will be decreased. It is possible to make changes to the tip or the flank. Profile changes are very important for high-load and high-speed gears. Other important parameters influencing the design of gears and their noise are the machining tolerances. With good tolerances, it is possible to obtain a remarkable reduction in noise, even for low-quality gears. However, it is necessary to remember that depending on the shape and model of the device, improvement of the teeth surface finish will lead to much higher costs, which can be more than half of the price of the device. Therefore, such expensive changes will not be easily accepted if they are simply for noise reduction purposes [26,27,28]. As mentioned previously, such toothed devices must have an appropriate backlash so that interferences during the meshing phase are avoided. In fact, tips and flanks that have too much backlash are very noisy because of the interactions between the teeth during no load or load inversion phases. For high-speed transmissions, even a small increase in the backlash can reduce the noise resulting from air and oil ejection—this often occurs in spur gear pairs, which are more likely to have noise problems. An added backlash on the root of a tooth helps to reduce the ejection speed, which can be achieved by increasing the total depth of the tooth. However, the benefits resulting from the reduction in noise must be calculated carefully because of the potential resistance loss for the teeth. However, when these sophisticated techniques are not appropriate from an engineering perspective, it is necessary to focus on the design of the case if we want to improve its dynamic response [29,30,31,32,33]. There are some important rules in this regard, such as avoiding big surfaces and small curvatures, since they vibrate uncontrollably if they are excited. Therefore, curvatures must be used only to stiffen the areas of the case that are characterized by high-energy stress intensity. If stiffening layers cannot be used, these sectors should have greater thicknesses so that they are more rigid than the other areas. However, this method has some restrictions, since a balance must be reached between the weight and project requirements. Therefore, the noise absorbed by the case system has to be evaluated carefully. In this report, through the mean analysis performed using the FEM program code, it was possible to describe how stresses resulting from meshing gears affected the acoustic emissions [34,35,36,37].

## 2. Materials and Methods

To perform the noise evaluation for the spur gears, FEM modeling and numerical simulation were performed. The first step in the FEM analysis was designing a spur gear pair. This was achieved using SolidWorks. The design parameters are shown in the following table (Table 1).

### FEM Static Analysis of a Spur Gear Pair

**Figure 1:**
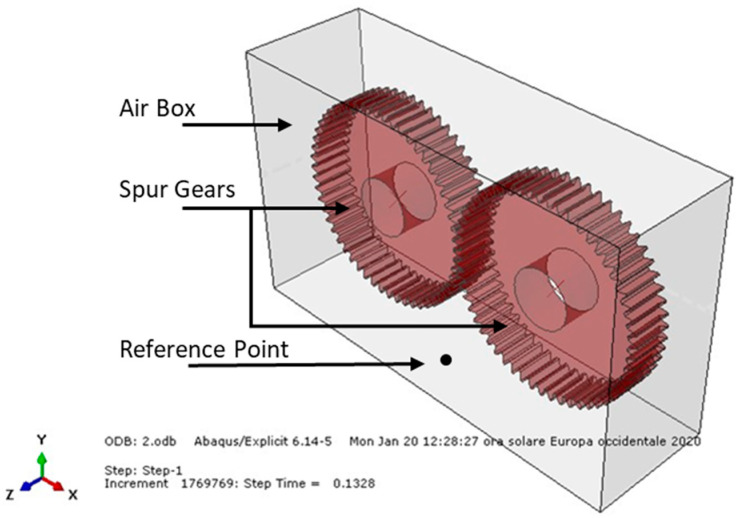
Spur gear assembly model geometry (isometric view).

To perform the FEM static analysis of the spur gear pair, the Abaqus/CAE 6.14-5 program code was used. The considered model was discretized using C3D8 brick elements, giving an 8-node linear brick (Abaqus Manual). The following picture (Figure 1) shows the modeled spur gears.

Regarding the FEM static analysis of the spur gear pair, five conditions were considered in order to evaluate the stress resulting from the meshing of the gears, since their acoustic emissions are influenced by the stresses resulting from the contact point [38,39]. Different parameters were taken into account for each condition considered, as shown in Table 2.

**Figure 2:**
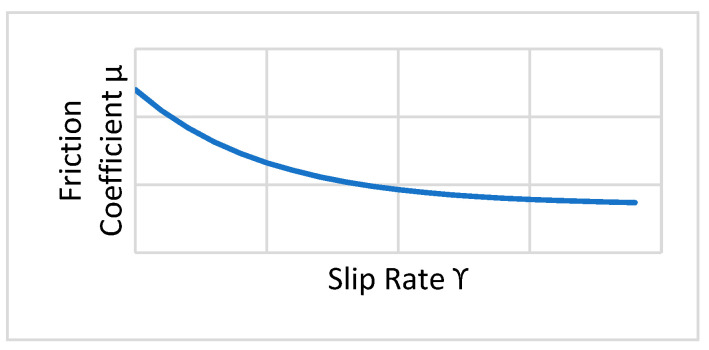
Exponential decay friction model.

An exponential decay friction model (*µ*) was adopted (Figure 2). It was necessary to insert the static friction coefficient *μ_s_*, the kinetic friction coefficient *μ_k_*, and the decay coefficient *d_c_* as well. This model was based on the following equation, where ϒ is the slip rate:

(1) $$\mu =\mu_{k}+\left(\mu_{s}-\mu_{k}\right)e^{-d_{c}ϒ}$$

## 3. Results

**Figure 3:**
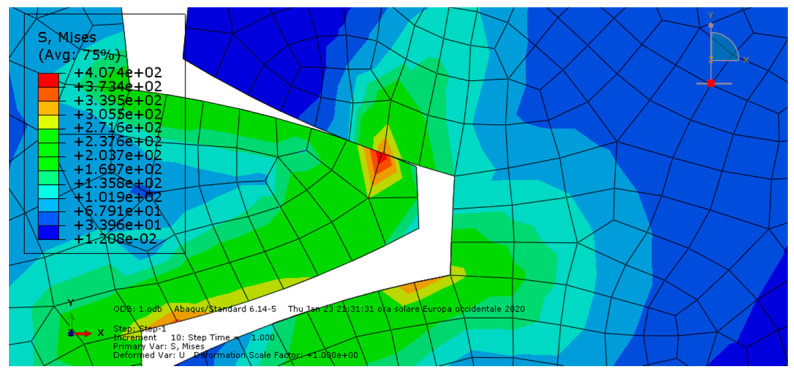
Condition A: The von Mises stress at the contact.

**Figure 4:**
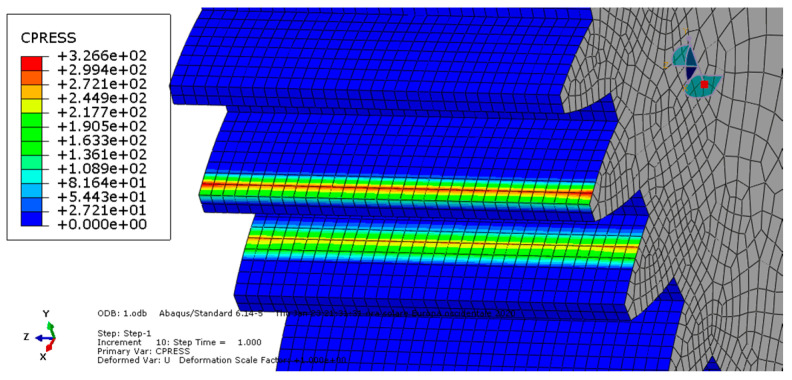
Condition A: Contact pressure along the tooth.

**Condition A**: The first analysis involved a steel spur gear pair and was characterized by frictionless contact. The results are shown in Figure 3 and Figure 4.

As we can see, the maximum von Mises stress equaled 407 MPa, while the contact pressure on the tooth amounted to 327 MPa.

**Figure 5:**
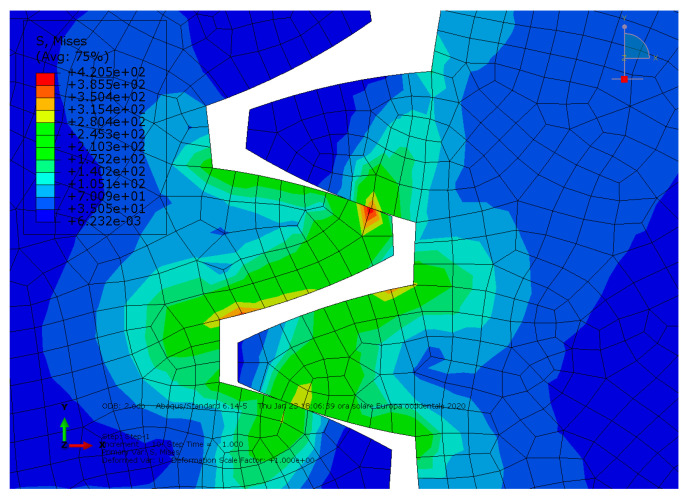
Condition B: The von Mises stress at the contact.

**Figure 6:**
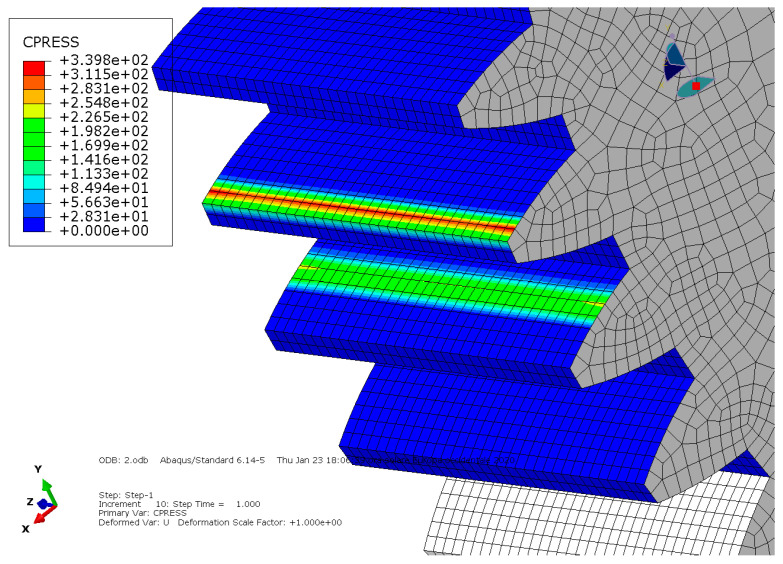
Condition B: Contact pressure along the tooth.

**Condition B**: The second analysis involved a steel spur gear pair and was characterized by the mentioned exponential-decay-based friction model. The results are shown in Figure 5 and Figure 6.

**Figure 7:**
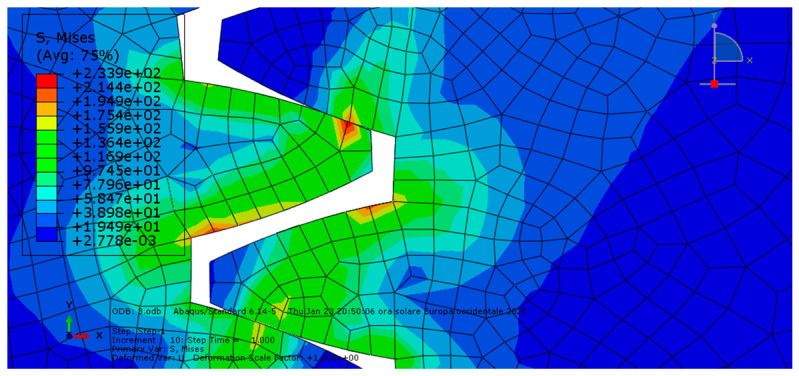
Condition C: The von Mises stress at the contact.

**Figure 8:**
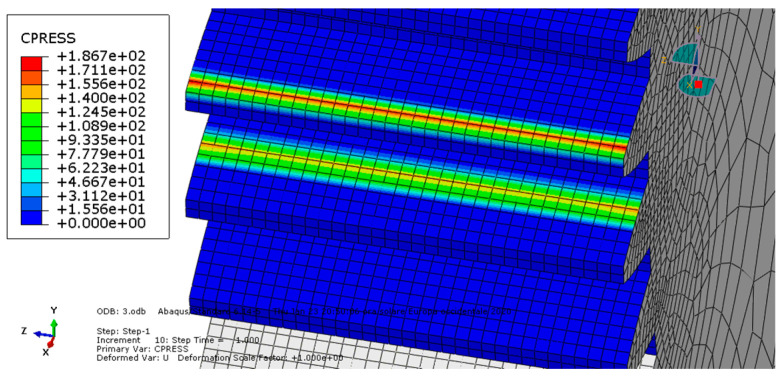
Condition C: Contact pressure along the tooth.

**Condition C:** The third analysis involved a ductile iron spur gear pair and was characterized by frictionless contact. The results are shown in Figure 7 and Figure 8.

As we can see, the maximum von Mises stress equaled 234 MPa, while the contact pressure on the tooth amounted to 187 MPa.

**Figure 9:**
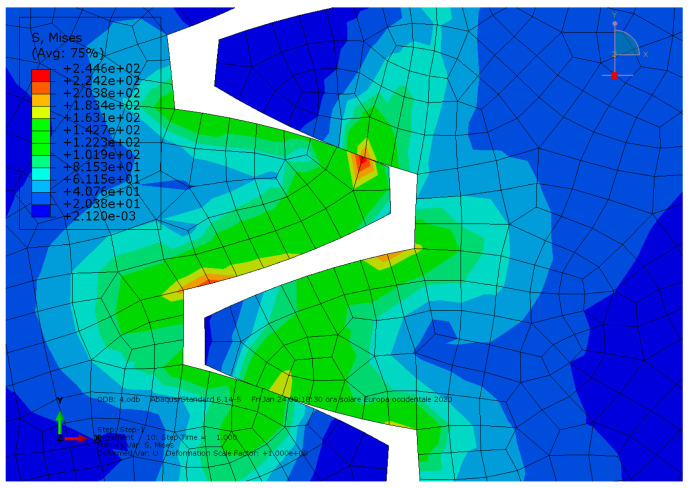
Condition D: The von Mises stress at the contact.

**Figure 10:**
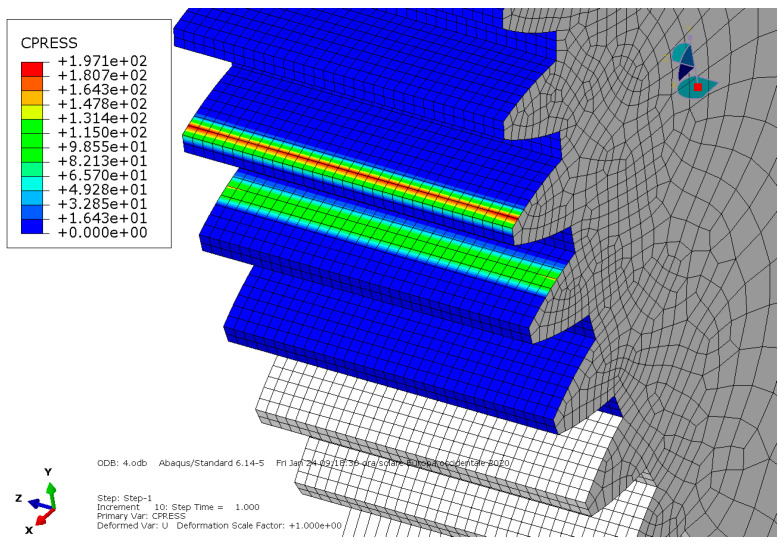
Condition D: Contact pressure along the tooth.

**Condition D**: The fourth analysis involved a ductile iron spur gear pair and was characterized by the mentioned exponential-decay-based friction model. The results are shown in Figure 9 and Figure 10.

As we can see, the maximum von Mises stress equaled 245 MPa, while the contact pressure on the tooth amounted to 197 MPa.

**Figure 11:**
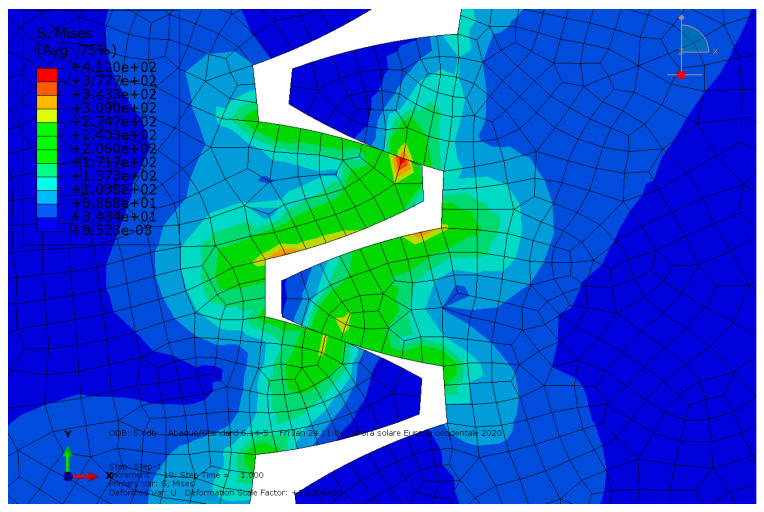
Condition E: The von Mises stress at the contact.

**Figure 12:**
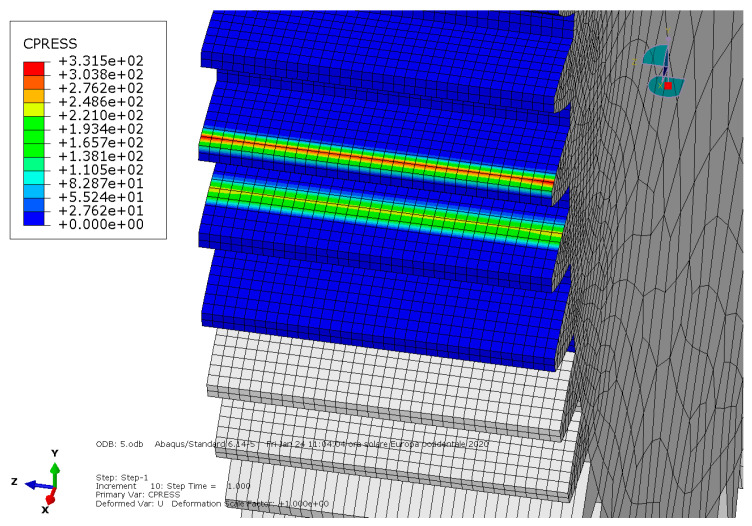
Condition E: Contact pressure along the tooth.

**Condition E**: The fifth analysis involved a steel spur gear pair and was characterized by the mentioned exponential-decay-based lubricated friction model. The results are shown in Figure 11 and Figure 12.

As we can see, the maximum von Mises stress equaled 412 MPa, while the contact pressure on the tooth amounted to 332 MPa.

### 3.1. Comparisons

First Analysis (Condition A) versus Second Analysis (Condition B)

The second analysis (condition B) showed higher von Mises stress and contact pressure values than the first one, so the presence of friction made the stress increase together with the sound excitation, as previously mentioned for this parameter.

Third Analysis (Condition C) versus Fourth Analysis (Condition D)

As we can see in the third and fourth analyses, the ductile iron spur gears had lower von Mises stress and lower contact pressure values, in agreement with comments regarding gear materials and ductile iron properties. Therefore, the sound excitation was lower as well.

Fifth Analysis (Condition E) versus First Analysis (Condition A)

The fifth analysis showed that lubricated friction made the stress decrease together with the contact pressure, leading to agreement that the oil coating reduced the sound excitation of the gears.

First Two Analyses (Conditions A and B) versus Third and Fourth (Conditions C and D) Analyses

The first two analyses (conditions A and B), which involved steel spur gear pairs, showed higher stress values than the third and fourth analyses, which involved ductile iron spur gears. These results again validated the previously mentioned theories about noise in gears.

**Figure 13:**
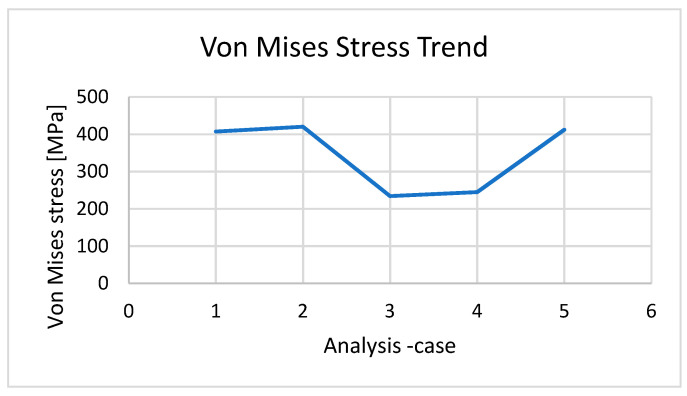
The von Mises stress trends between the different analyses.

In Figure 13, the diagram shows the von Mises stress trends between the different analyses.

**Figure 14:**
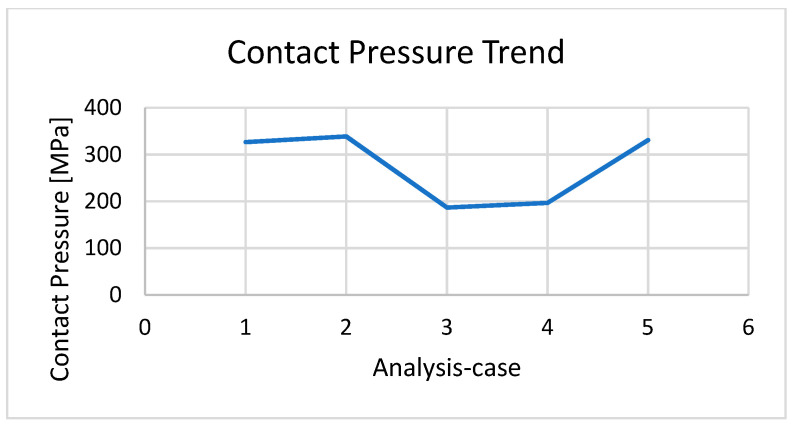
Contact pressure trends between the different analyses.

In Figure 14, the diagram shows the contact pressure trends between the different analyses.

Finally, we can see that the contact pressure distribution was correct, which allowed us to say that the analyses were carried out in a proper and accurate manner, providing reliable results.

### 3.2. Difference Static and Dynamic Analyses

The static analyses were mainly carried out with the aim of setting all of the basic parameters necessary for the simulations (the type of material, friction, constraint conditions, and contact algorithms used). Once all of the basic parameters had been appropriately set (by checking for the presence of any penetration and assessing that the distribution and stress values were acceptable), the model was further complicated by inserting an airbox and carrying out coupled Eulerian–Lagrangian (CEL) analyses [40,41,42]. The parameters that affected this second type of analysis were related to the evaluation of the acoustic emissions, for which we focused on the friction, lubrication, and rotation speed. The second type of material was not included among the variables, as the required number of simulations involving analyses requiring long computation times would have greatly increased [43,44].

### 3.3. Coupled Eulerian–Lagrangian (CEL) Analysis of a Spur Gear Pair

CEL analyses are very demanding in terms of the computation time; therefore, the box sizes were as small as possible in order to contain the gear wheels and limit the number of elements. 

Constraint conditions were applied to avoid any disturbing effects; for example, waves could be reflected on the edges of the box walls. Indeed, the air domain was assumed to be practically infinite. The keyword used in the Abaqus input file that allowed this condition to be achieved was:

“EULERIAN BOUNDARY, OUTFLOW = NON REFLECTING”.

Within the air domain, a node was chosen near the walls in order to measure the sound pressure level. Its exact position was 80 mm below the midpoint of the wheelbase of the two wheels. Both the distance of 80 mm as that of a meter has no particular meaning because they are used only as reference points to study the sound variations [45,46,47]. These values were only set as references to allow a comparison between the various analyses under equivalent conditions. The pressure values obtained from these analyses were then extrapolated to a distance of 1 m (with the following formula) with respect to the reference point (Figure 1).

(2) $$SPL=SPL_{ref}-20log_{10}\left(\frac{r}{r_{ref}}\right)$$

where *SPL* is the sound pressure level at a distance of 1 m; *SPL_ref_* is the sound pressure level at a distance of about 80 mm; *r* is a distance of 1 m; *r_ref_* is a distance of about 80 mm.

In this way, in order to evaluate the noise level deriving from the meshing spur gear pair, the pair was modeled with a Lagrangian formulation. The gears were in contact during their operation and were considered to be “immersed” in an air box (modeled in an Eulerian way) in order to allow the propagation of mechanical and acoustic waves inside the considered mean. 

The airbox dimensions were as follows: X = 350 mm; Y = 200 mm; Z = 100 mm. The airbox was modeled with EC3D8R brick elements; that is, an 8-node linear Eulerian brick, reduced integration, and hourglass control (Abaqus Manual). The number of Eulerian elements used was 155,382. Therefore, in order to evaluate the sound pressure level resulting from the meshing spur gear pair, different coupled Eulerian–Lagrangian analyses were carried out, putting the gears in an Eulerian mesh, which represented air (modeled as a perfect gas), since noise is characterized by pressure waves that propagate through a medium. A noise, vibration, and harshness (NVH) analysis could not be properly performed, since it could only be used to evaluate the noise resulting from rolling bearings and supporting system vibrations [48,49,50,51]. The following table (Table 3) shows the parameters that characterized the different examined conditions.

As explained in [1], air was modeled as an ideal gas and entropy variation was imposed as zero (*ds* = 0). Despite friction being taken in account, no energy degradation was considered, meaning the temperature remained constant and equal to room temperature.

**Figure 15:**
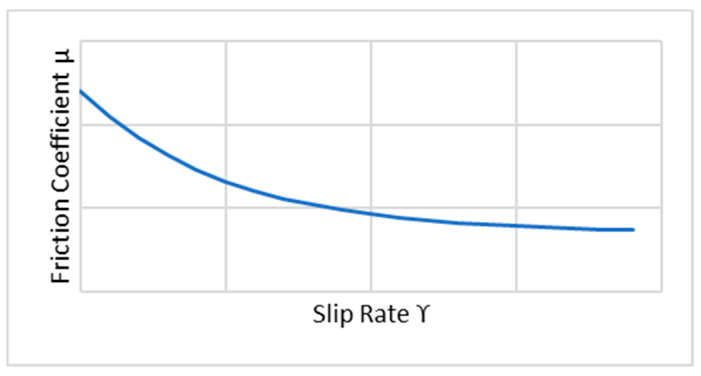
Exponential decay friction model.

Regarding the friction model, the same exponential decay friction model was used. It was necessary to insert the static friction coefficient *μ_s_*, the kinetic friction coefficient *μ_k_*, and the decay coefficient *d_c_* as well. This model was based on Equation (1), in which ϒ is the slip rate (Figure 15).

Finally, the sound pressure level resulting from gear meshing was evaluated at a distance of 1 m thanks to Equation (2).

## 4. Discussion

**Figure 16:**
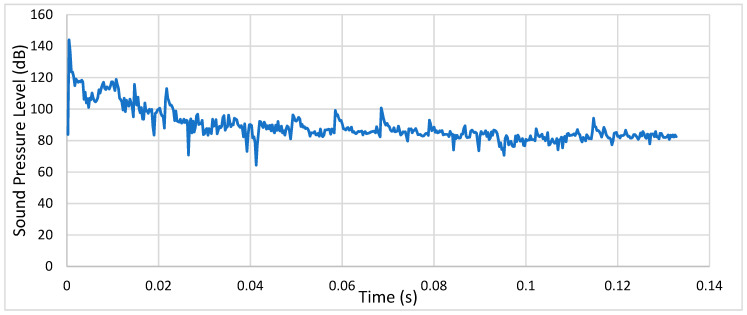
Condition 1: Sound pressure level spectrum.

**Condition 1**: The first analysis involved a steel spur gear pair and was characterized by frictionless contact and a rotational speed of 500 RPM. Figure 16 shows the sound pressure level of the meshing gears at a distance of 1 m.

As we can see, after the initial transient peak, the SPL stabilized between 80 and 85 dB.

**Figure 17:**
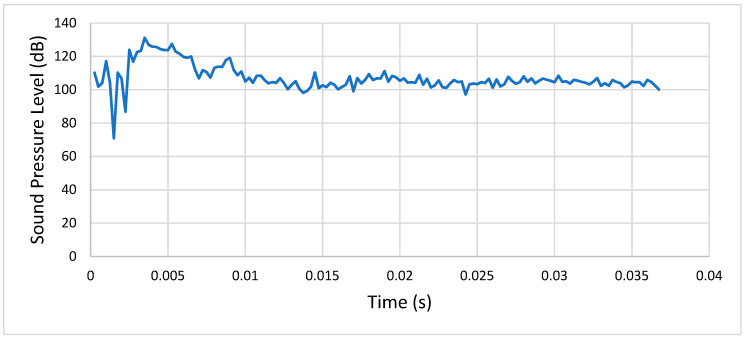
Condition 2: Sound pressure level spectrum.

**Condition 2**: The second analysis involved a steel spur gear pair and was characterized by the mentioned exponential decay friction model and a rotational speed of 500 RPM. Figure 17 shows the sound pressure level of the meshing gears at a distance of 1 m.

As shown, after the initial transient peak, the SPL stabilized at over 100 dB

**Figure 18:**
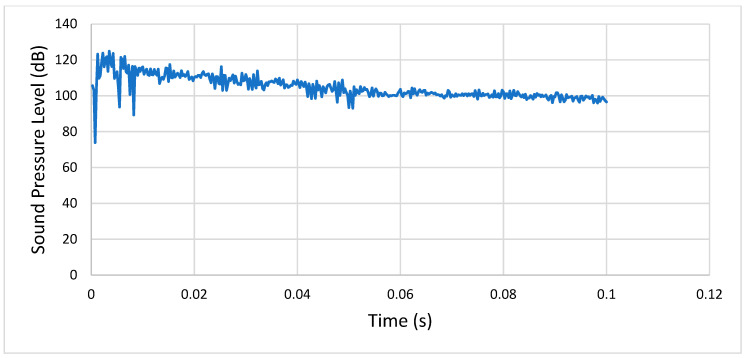
Condition 3: Sound pressure level spectrum.

**Condition 3**: The third analysis involved a steel spur gear pair and was characterized by the mentioned exponential decay friction model and a rotational speed of 1500 RPM. Figure 18 shows the sound pressure level of the meshing gears at a distance of 1 m.

As we can see, after the initial transient peak, the SPL stabilized at around 100 dB

**Figure 19:**
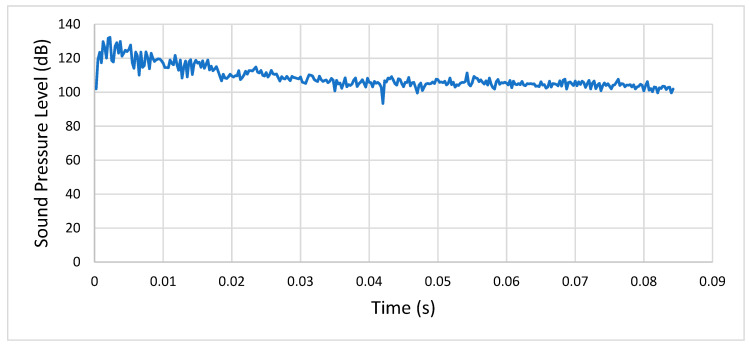
Condition 4: Sound pressure level spectrum.

**Condition 4**: The fourth analysis involved a steel spur gear and was characterized by the mentioned exponential decay friction model and a rotational speed of 3000 RPM. Figure 19 shows the sound pressure level of the meshing gears at a distance of 1 m.

As shown, after the initial transient peak, the SPL stabilized between 100 and 110 dB.

**Figure 20:**
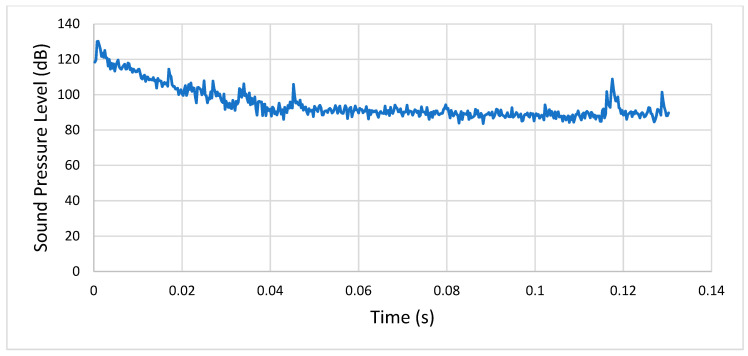
Condition 5: Sound pressure level spectrum.

**Condition 5**: The fifth analysis involved a steel spur gear pair and was characterized by the mentioned exponential decay friction model (lubricated friction) and a rotational speed of 500 RPM. Figure 20 shows the sound pressure level of the meshing gears at a distance of 1 m.

As we can see, after the initial transient’s peak, the SPL stabilized between 85 and 100 dB.

### 4.1. Comparisons

#### 4.1.1. First Analysis (Condition 1) versus Second Analysis (Condition 2)

**Figure 21:**
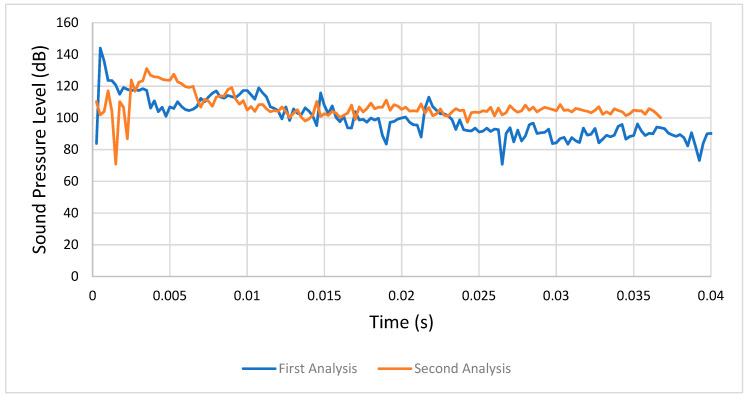
Comparison between the first (condition 1) and second analyses (condition 2).

In the first analysis, it was possible to see that the SPL stabilized between 80 and 85 dB, while in the second one it stabilized at over 100 dB because of the friction’s presence, which meant the gears’ acoustic emissions were higher. Therefore, we can agree with the comments about the influence of friction on gear noise. Figure 21 shows a comparison between the respective sound pressure levels of the first and second analyses.

#### 4.1.2. Third Analysis (Condition 3) versus Fourth Analysis (Condition 4)

**Figure 22:**
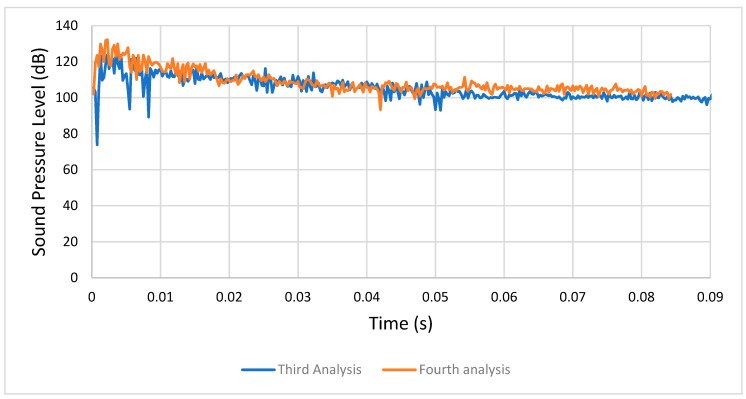
Comparison between the third (condition 3) and fourth analyses (condition 4).

In the third analysis, after the initial transient peak, the SPL stabilized at around 100 dB, while in the fourth one it stabilized between 100 and 110 dB, because of the increased rotational speed (it went from 1500 RPM to 3000 RPM), as well as the friction. Therefore, we can agree again with the idea that the increasing rotational speed affected the acoustic emission of the meshing gears. Figure 22 shows a comparison between the respective sound pressure levels from the third and fourth analyses.

#### 4.1.3. Fourth Analysis (Condition 4) versus Fifth Analysis (Condition 5)

**Figure 23:**
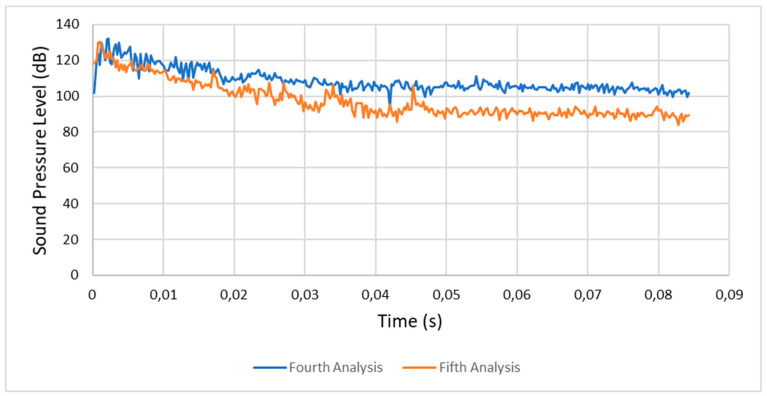
Comparison between the fourth (condition 4) and fifth analyses (condition 5).

In the fourth analysis the SPL stabilized between 100 and 110 dB, while in the fifth analysis it stabilized between 80 and 100 dB. This was due to the presence of lubricated friction and a lower rotational speed. Figure 23 shows a comparison between the respective sound pressure levels of the fourth and fifth analyses.

#### 4.1.4. Second Analysis (Condition 2) versus Fifth Analysis (Condition 5)

**Figure 24:**
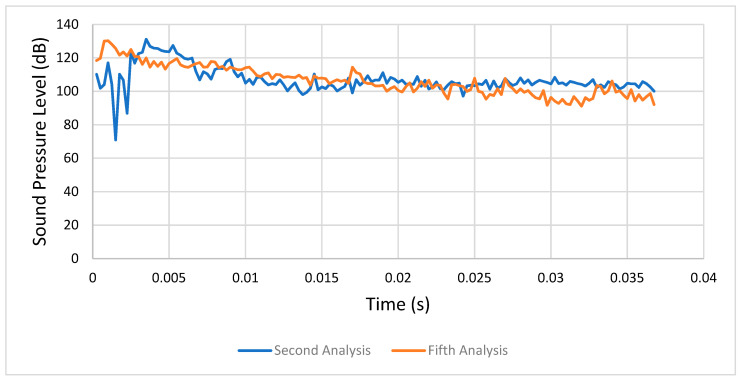
Comparison between the second (condition 2) and fifth analyses (condition 5).

In the second analysis the SPL stabilized at over 100 dB, while in the fifth analysis it stabilized between 85 and 100 dB. This was explained by the presence of lubricated friction in the fifth analysis, meaning we found a lower level of acoustic excitation. Figure 24 shows a comparison between the respective sound pressure levels of the second and fifth analyses.

#### 4.1.5. Final Remarks

In the model adopted for the CEL analysis, air was considered a perfect gas, but it could be changed by taking into account the viscosity of air or another state equation. Therefore, different results could have been obtained, leaving an opportunity to do more research in this field.

## 5. Conclusions

Noise reduction, at least in Europe, has become a major concern for communities [52,53]. This concern has led to great societal pressure on policy-makers, thus giving rise to stacked legislations and regulations at various levels. In Europe, two directives address noise issues, the first from a general standpoint and the second one specifically in regard to noise-related operating restrictions at the community level. Both of these EC directives refer to notions that are now commonly handled by specific industries, such as noise mapping or dose–response curves. Each of the aforementioned regulations has triggered an ongoing effort by engine manufacturers and by their associated research centers to define and follow a path toward more silent devices. 

In this work, the acoustic emissions generated due to the spur gears meshing was studied and various values of the operating parameters were considered. The work was organized into two stages: (1) static analysis of the meshing gears was conducted to set and evaluate the goodness of the parameters of the model and to study the stress levels reached in the teeth; (2) coupled Eulerian–Lagrangian (CEL) analysis was performed to evaluate the noise generation. In this stage, the spur gears were “immersed” in an air box at room temperature. In this way, the pressure waves generated due to the interactions of the gears could propagate up to a reference point, where the sound pressure level was measured. Many work conditions were taken into account in the second stage in order to study how they can affect noise generation. The air was modeled as an ideal gas at room temperature and no heat flux was generated due to the friction among the gears. The entropy was set as constant (ds = 0) and an adiabatic process was considered. The results were in good agreement with the literature. 

Future developments in this area could include analysis of how the heat generated during the meshing affects the noise generation. In this way, a damage detection model that considers the interactions between the noise generation and the entropy formation could be developed and tested on an experimental apparatus.

## Figures and Tables

**Table 1:** Parameters and corresponding values for the spur gears.

Parameters	Values
Number of teeth N	55
Module	2.72
Pitch circle	150 mm
Dedendum circle	142.50 mm
Addendum circle	156 mm
Circular pitch	6.5°
Pressure angle	20°

**Table 2:** Parameters of the conditions considered.

Condition	Friction	Material Parameters	Lubricated Friction
A	NO	Steel	NO
B	*μ_s_* = 0.74 *μ_k_* = 0.57 *d_c_* = 0.2	Steel	NO
C	NO	Ductile Iron	NO
D	*μ_s_* = 1.1 *μ_k_* = 0.15 *d_c_* = 0.2	Ductile Iron	NO
E	NO	Steel	*μ_s_* = 1.1 *μ_k_* = 0.15 *d_c_* = 0.2

**Table 3:** Parameters of the analysis.

Condition	Parameters			
	Friction	Material	Lubricated Friction	Rotational Speed (RPM)
1	NO	Steel	NO	500
2	*μ_s_* = 0.74 *μ_k_* = 0.57 *d_c_* = 0.2	Steel	NO	500
3	*μ_s_* = 0.74 *μ_k_* = 0.57 *d_c_* = 0.2	Steel	NO	1500
4	*μ_s_* = 0.74 *μ_k_* = 0.57 *d_c_* = 0.2	Steel	NO	3000
5	NO	Steel	*μ_s_* = 0.11 *μ_k_* = 0.05 *d_c_* = 0.2	500
